# Complete Genome Sequence of *Thalassospira* sp. Strain GO-4, a Marine Bacterium Isolated from a Phenanthrene-Enriched Bacterial Consortium

**DOI:** 10.1128/mra.00532-22

**Published:** 2022-07-07

**Authors:** Go Kayama, Robert A. Kanaly, Jiro F. Mori

**Affiliations:** a Graduate School of Nanobiosciences, Yokohama City University, Yokohama, Japan; University of Southern California

## Abstract

The genus *Thalassospira* has often been studied as a potential major contributing member of aromatic hydrocarbon-exposed microbial communities. Here, the complete genome sequence of a new isolate of *Thalassospira*, strain GO-4, was obtained and was confirmed to possess functional genes that are responsible for its metabolism of phthalic acid.

## ANNOUNCEMENT

A new isolate of *Thalassospira*, strain GO-4, was obtained from a phenanthrene-enriched marine bacterial consortium. This consortium was sampled from the coast of Nojima, Yokohama, Japan (35.328520N, 139.636326E), in December 2019. Bacterial cells were collected by filtering 1 L of coastal seawater through a 0.22-μm Rapid-Flow filter (Thermo Fisher Scientific, Waltham, MA), and the filter was used as the inoculant for a routinely transferred bacterial enrichment culture that was supplied with 50 mg L^−1^ phenanthrene as the carbon source in artificial seawater (ASW) medium (1). Strain GO-4 was isolated from this consortium through dilution-to-extinction methods with 10 mM glucose as the carbon source. *Thalassospira* species have often been identified as members of aromatic hydrocarbon-exposed microbial communities (2–5) and are considered to contribute to aromatic hydrocarbon biodegradation (6–8). Therefore, to expand our understanding of the metabolic potential of *Thalassospira*, the complete genome of strain GO-4 was sequenced and its functional genes were investigated.

The complete genome of strain GO-4 was obtained through hybrid assembly of DNBSEQ-G400 short-read (MGI Tech, Shenzhen, China) and GridION long-read (Oxford Nanopore Technologies, Oxford, UK) sequencing data. The genomic DNA of strain GO-4 was extracted using the NucleoBond high-molecular-weight DNA kit (Macherey-Nagel, Germany) after culturing for 6 days on 10 mM glucose in ASW medium. The DNBSEQ library was prepared with the MGIEasy FS DNA library preparation set (MGI Tech). A total of 22,517,128 reads with 200-bp paired-end read lengths were obtained; they were then trimmed and quality filtered using Cutadapt (v4.0) (9), SeqKit (v0.13.2) (10), and Sickle (v1.33) (11). The GridION library was created using the ligation sequencing kit SQK-LSK109 (Oxford Nanopore Technologies) (>3-kb fragments were enriched by the protocol). A total of 68,243 reads (average length, 17,703 bp) were generated by the GridION platform using R9.4.1 flow cells and Guppy (v4.0.11) (12) for live base calling. Raw reads were subjected to trimming and quality filtering using Porechop (v0.2.3) (13) and Filtlong (v0.2.0; minimum length of 1,000 bp) (https://github.com/rrwick/Filtlong). *De novo* hybrid assembly was performed using Unicycler (v0.4.7) (14) to assemble these sequencing data, and Bandage (v0.8.1) (15) was used to validate the assembled sequence. All software was used with default settings unless otherwise indicated.

The complete genome sequence thus obtained consisted of a single circular chromosome (4,546,452 bp, with 1,256× coverage and a G+C content of 54.8%) without plasmids. According to the NCBI Prokaryotic Genome Annotation Pipeline (PGAP) (v6.1), this chromosome carried 4,046 coding sequences (CDSs), 12 rRNAs, and 64 tRNAs.

The strain GO-4 genome possessed homologs of known functional genes that are responsible for phthalic acid and protocatechuic acid biodegradation, i.e., *pht* genes (16) and *pca* genes (17), respectively. This suggests that *Thalassospira* contributes to the biotransformation of phthalic acid and structurally related aromatics in the bacterial consortium from which it originated.

### Data availability.

The genome sequence of strain GO-4 is available in NCBI GenBank under the accession number CP097807. The raw sequence data are also available with the SRA accession numbers SRR19369649 and SRR19369650, under BioProject accession number PRJNA841389 and BioSample accession number SAMN28591689.
